# The Effects of a Body Safety Training Program for Children from Low-Income Families on Children’s Self-Protection Skills

**DOI:** 10.3390/healthcare14162607

**Published:** 2026-08-19

**Authors:** Mehtap Genç, Fatma İzciler, Nisa Aslan

**Affiliations:** 1 Department of Mental Health and Psychiatric Nursing, Faculty of Health Sciences, Karamanoğlu Mehmetbey University, 70200 Karaman, Türkiye; 2 Department of Nursing, Faculty of Health Sciences, Karamanoğlu Mehmetbey University, 70200 Karaman, Türkiye; fatmaizciler@gmail.com (F.İ.); nisasl12345a@gmail.com (N.A.)

**Keywords:** child sexual abuse, body safety, self-protection skills, low-income families, preschool children

## Abstract

**Background:** Child sexual abuse is a major public health concern, particularly for children from socioeconomically disadvantaged families. This study examined the effect of a body safety training (BST) program on the self-protection skills of preschool children from low-income families. **Methods:** This one-group pretest–posttest quasi-experimental study was conducted with 32 children aged 4–6 years attending a public kindergarten located in a low-income neighborhood in Türkiye. The children participated in a seven-session BST program. Data were collected via the What If Situations Test (WIST), with assessments conducted before and after the intervention. Differences between the pretest and posttest scores were analyzed via the Wilcoxon signed-rank test, and effect sizes (r) were calculated. **Results:** Statistically significant improvements were observed in the WIST subscales of appropriate touching recognition, inappropriate touching recognition, Saying skill, Doing skill, Telling skill, and Reporting skill, as well as in the Personal Safety Questionnaire scores (all *p* < 0.05). A significant increase with a large effect size was observed in the WIST total skill scores following the intervention (Z = −4.756, *p* < 0.001, r = 0.84; mean change = 6.34, 95% CI: 4.63–8.13). **Conclusions:** Participation in the BST Program was associated with significant improvements in the self-protection skills of preschool children from low-income families. These findings suggest that structured body safety education may be a promising approach for supporting personal safety skills among socioeconomically disadvantaged preschool children.

## 1. Introduction

Child abuse and neglect represent global public health issues observed across generations, socioeconomic groups, and societies [1]. Among all forms of abuse, child sexual abuse has the most devastating consequences and constitutes a severe violation of human rights [1,2]. Millions of children and adolescents worldwide experience sexual abuse, including sexual assault or rape [2]. Finkelhor et al. (2015), in their large-scale study involving 4000 children and adolescents aged 0–17, revealed that 5% of participants were exposed to sexual offenses and that 1.4% were exposed to sexual assault in the past year [3]. Child sexual abuse, including many forms of sexual harassment against children, such as sexual assault, rape, incest, and commercial sexual exploitation, is associated with a range of adverse outcomes [4]. Child sexual abuse is associated with high risks of psychosocial, psychiatric, and physical health outcomes, including sexually transmitted infections; HIV infection; unintended pregnancies; gynecological diseases; physical and psychological trauma; engagement in high-risk sexual behavior later in life; substance abuse; conversion disorder; borderline personality disorder; schizophrenia; depression; anxiety; posttraumatic stress disorder; externalization symptoms; sleep disorders; and a high risk of suicidal and self-harming thoughts [1,2,5,6,7].

The high incidence of child sexual abuse, together with its short- and long-term adverse health consequences, makes the prevention of these crimes a societal priority [8,9]. One of the most commonly used methods in this field includes teaching school-aged children about sexual abuse and ways to protect themselves through school-based programs [10]. School-based and preschool programs such as the Body Safety Training (BST) Program, Parents as Teachers of Safety (PaTS), Safe Touches: Personal Safety Training, Safer, Smarter Kids, and Talking About Touching have several strengths, including enhancing children’s personal safety skills, increasing their knowledge of child sexual abuse, and fostering communication [11]. The results from numerous studies employing different methodologies have also shown that school-based child abuse prevention programs are effective in increasing children’s knowledge levels and self-protection skills [10,12,13,14,15,16]. In a randomized controlled study conducted with 1176 students in Florida, the effectiveness of a curriculum program designed to educate children about bullying, cyberbullying, and four types of maltreatment (physical, sexual, emotional abuse, and neglect) was evaluated. Notably, the results revealed that children who participated in the program increased their knowledge about potentially risky situations, and these improvements persisted in follow-up assessments conducted seven months later [17]. Rudolph and Zimmer-Gembeck (2018) also reported that programs targeting children increased their knowledge about sexual abuse and their self-protection skills [8]. A recent meta-analysis by Gubbels et al. (2021) revealed that interventions implemented in daycare centers or preschools had a greater impact on self-protection skills than did primary school interventions [13].

Child sexual abuse is widely recognized as a complex phenomenon shaped by multiple causes, expressed in different forms, and embedded in various relationships across families, peer groups, institutions, and communities [18]. Therefore, sexual abuse crimes against children occur across heterogeneous populations and represent a multifaceted problem [9]. Low economic income is considered one of the family-related risk factors (e.g., fragmented family structures, neglectful and indifferent parenting attitudes, lack of love and affection within the family, violence, communication problems, alcohol and drug use) for child sexual abuse [19]. A prospective study with a large sample (*n* = 1087) of girls also demonstrated that socioeconomic status is an important risk factor for child sexual abuse [20]. A previous study revealed that the risk of sexual assault decreases as the level of economic wealth increases, whereas girls raised in families at or below the poverty line are at greater risk of victimization [20]. Recent evidence suggests that socioeconomic context may influence children’s baseline CSA-related knowledge and the outcomes of school-based prevention programs, whereas children across different socioeconomic backgrounds can benefit from such interventions [21,22].

Although previous studies generally support the benefits of school-based child sexual abuse prevention programs, the available evidence is heterogeneous in terms of children’s age, socioeconomic background, intervention content, and study design. In particular, studies specifically examining body safety interventions among preschool children from low-income families remain limited. Therefore, further research focusing on this socioeconomically vulnerable preschool population is needed. The aim of this study was to examine changes in children’s self-protection skills following their participation in the BST Program. The research question was whether children’s self-protection skills differed between the pretest and posttest assessments following their participation in the BST Program.

## 2. Materials and Methods

### 2.1. Research Design

In this study, a single-group pretest–posttest quasi-experimental research study design was employed.

### 2.2. Research Variables

The independent variable of this study was the BST Program, whereas the dependent variable was the score obtained from the What If Situations Test (WIST).

### 2.3. Population and Sample

The study was conducted with children attending a public kindergarten located in a neighborhood inhabited by low-income families in a province located in the Central Anatolia Region of Turkey. The school is located in an urban area of the city and is known for its low socioeconomic status and high rates of violence. A total of 71 children (aged 4–6 years) enrolled in kindergarten during the 2023–2024 academic year constituted the study population. On the basis of a power analysis conducted with the GPower version 3.1 (Heinrich Heine University Düsseldorf, Düsseldorf, Germany)program, with an effect size of 0.60 (large effect), an error level (α) of 0.05, and a test power (1–β) of 0.90, the minimum sample size was determined to be 26.

The inclusion criteria for the study were being between the ages of 3–6, having no Smental or communicative disability that would prevent them from answering the questions in the data collection form, speaking Turkish, having parental consent, and having parents reporting a low-income status. The exclusion criterion was failure to meet the inclusion requirements. Three children were excluded because of speech difficulties, and one child was excluded because of autism spectrum disorder. Among the remaining 67 children, 32 whose parents provided written consent and who volunteered to participate in the study composed the sample.

### 2.4. Intervention: I Am the Boss of My Own Body: A Body Safety Training Program for Preschool Children

The BST Program, originally developed in 1986 by Sandy K. Wurtele, was revised in 2007 to its current form. Designed for preschool-aged children (3–6 years), the program aims to prevent child sexual abuse and enhance children’s self-protection skills. The original version consists of 10 group-based sessions [23]. Its Turkish adaptation was carried out by Çıtak Tunç (2018), in which the program was modified into seven sessions [24]. Additionally, an educational book entitled “I Am the Boss of My Body—A Body Safety Training Program for Preschool Children” was prepared for use during the sessions [24]. The book illustrates the stories used in the training, containing a total of 72 images. These visual materials are used to narrate stories during the program, and children are asked questions related to the stories as part of the learning process. The BST program consisted of seven sessions, each focusing on specific body safety objectives and skills. Session 1 introduced body ownership and basic body safety concepts. Session 2 focused on recognizing and responding to potentially unsafe situations involving strangers, including refusing gifts, avoiding entering a stranger’s car, and distinguishing safe from potentially dangerous places. Session 3 introduced private parts and appropriate situations in which another person may look at or touch them. Session 4 focused on distinguishing appropriate from inappropriate situations involving private parts and reinforced body safety rules through stories and the “right–not right” game. Session 5 introduced and practiced key self-protection skills, including saying “No,” getting away from an unsafe situation, and telling a trusted person. Session 6 reinforced disclosure and reporting skills, including continuing to tell until someone helps, practicing how to report an incident, and emphasizing that inappropriate touching or abuse is never the child’s fault. Finally, Session 7 consolidated the program through the practice and reinforcement of the body safety and self-protection skills learned in the previous sessions [24].

Each session lasts approximately 15–20 min and is conducted in a group format. The ideal group size is reported to be between six and ten children, with recommendations to reduce the number of participants as the children’s age decreases [25]. This study employed the educational book “I Am the Boss of My Body: A Body Safety Training Program for Preschool Children”, containing the seven-session training program developed by Çıtak Tunç (2018) [24]. M.G. completed the in-person “Body Safety Training for Practitioners in Preventing Sexual Abuse” course, conducted by Dr. Gülseren Çıtak Tunç on 13 July 2019, prior to implementing the intervention. The BST program was delivered according to the planned session content and structure; however, no formal intervention fidelity monitoring, such as an independent observer or standardized fidelity checklist, was conducted.

### 2.5. Procedure

The data collection and implementation process was completed in February 2024. Following the permissions required to conduct the study, the first step involved informing the administrators and teachers of the relevant kindergarten about the aim and content of the study. During the parent meeting held in February 2024, researchers, with the cooperation and supervision of administrators and teachers, informed parents about the aim and content of the study. Afterward, the written and verbal consent of parents who agreed to include their children in the education program was obtained, and parents completed the ‘Parent–Child Information Form’. All the children who provided written consent from their parents were enrolled in the training program and assigned to 5 groups of 6–7 children, as recommended in the intervention guidelines. The data collection and training sessions were completed face-to-face in a distraction-free room provided by the school administration during time slots designated by the administrators and teachers. Before starting the training program, the children were introduced to the researchers. On the first day, a pretest (the What If Situations Test) was administered. Sessions were completed over the following 7 consecutive days (excluding weekends). One day after the final session (7th session), a posttest was administered to the children. Each session lasted approximately 20 min. The pretest and posttest outcome assessments were conducted by researchers who were also involved in implementing the BST Program; therefore, the outcome assessors were not blinded to the assessment time points.

### 2.6. Instruments

Parent–Child Information Form: The parent–child information form, prepared by researchers, consists of 11 questions assessing the sociodemographic characteristics of the child (age, gender, number of siblings, presence of physical or mental illness, number of people living in the household in addition to the child) and the parent (age, gender, educational status, occupation, family type, income status).

What If Situations Test (WIST): Developed by Wurtele et al. (1998), the WIST was designed to assess preschool children’s self-protection skills in situations involving sexual abuse [26]. The Turkish adaptation of the test was conducted by Çıtak Tunç (2018) [27]. The instrument consists of eight short stories, of which the first two are practice items and are not included in scoring. The remaining six stories are used to measure children’s self-protection skills. The test includes the subscales “Appropriate Touch Recognition” and “Inappropriate Touch Recognition,” which assess the child’s ability to distinguish between appropriate and inappropriate touch. In the Appropriate Touch Recognition subscale, children listen to stories and are expected to respond correctly that, in necessary situations, a mother, father, doctor, or nurse may touch or examine private parts. A “yes” answer is given a score of 1 point, whereas “no” or “don’t know” responses are given a score of 0 points. In the stories of the Inappropriate Touch Recognition subscale, children are expected to provide an answer indicating that it is not right for a familiar or unfamiliar person to touch or look at the child’s private parts in inappropriate situations. A “no” answer is given a score of 1 point, whereas “yes” or “don’t know” responses are given a score of 0 points. For each inappropriate-touching scenario, the child’s responses to the following questions are evaluated: “What would you say?”, “What would you do?”, “Would you tell someone? Who would you tell?”, and “What would you say to this person in such a situation?” The responses to these questions are evaluated in terms of four skill scores: Say, Do, Tell, and Report. Each response is scored from 0 to 2 according to the WIST scoring criteria. For Say Skill, definite verbal refusal is given 2 points, tentative verbal refusal is given 1 point, and no verbal refusal is given 0 points. For Do Skill, immediate and definite escape or refusal is given 2 points, vague or delayed escape or refusal is given 1 point, and no escape or refusal is given 0 points. For Tell skill, identifying two or more people to tell is scored as 2 points, identifying one person as 1 point, and identifying no one as 0 points. For Report Skill, correctly identifying both the person involved and the situation is scored as 2 points, identifying either the person or the situation as 1 point, and providing no accurate information as 0 points. The three inappropriate-touch scenarios are used to calculate each of the four skill subscale scores. All four skill domains were scored for each child across the three inappropriate-touching scenarios, with inappropriate or absent responses scored as 0; therefore, the Say, Do, Tell, Report, and Total Skill scores were calculated for the entire sample (*n* = 32). Accordingly, the Say, Do, Tell, and Report skill subscale scores each range from 0–6 points, and the total skill score, obtained by summing these four skill scores, ranges from 0–24 points. The final subscale of the test is the “Personal Safety Questionnaire” (PSQ), which was subsequently added as complementary items to the WIST. The PSQ subdimension, consisting of two stories and four questions, assesses the child’s level of knowledge about sexual abuse and attitudes toward sexuality. The PSQ score is calculated on a scale of 0–4 points on the basis of the “yes,” “no,” and “I don’t know” responses to the questions. The Cronbach’s alpha reliability coefficient for the Turkish version of the test was reported to range between 0.69 and 0.90 [27]. In our study, the Cronbach’s alpha reliability coefficient for the WIST was found to be 0.727 for the pretest and 0.767 for the posttest.

### 2.7. Data Analysis

Data were analyzed via the IBM SPSS Statistics for Windows, version 28.0 (IBM Corp., Armonk, NY, USA). There were no missing data for the study variables; therefore, no imputation or other missing-data procedures were needed. To assess the normality of the scale scores, the skewness and kurtosis values were examined, with the acceptable range for a normal distribution set at ±1.5 [28]. Since the data did not meet the normality assumption, the Wilcoxon signed-rank test was employed to compare the pretest and posttest scores. In addition, mean pretest–posttest differences and their 95% confidence intervals (CIs) were estimated via paired-samples *t*-tests with 5000 bootstrap samples. Effect sizes (r) were calculated to determine the magnitude of significant differences, and the corresponding cutoff values and interpretations are presented in the relevant subsection. In addition, the relationships between children’s posttest scores on the WIST-Total Skill and the Personal Safety Questionnaire were analyzed via Spearman’s rank–order correlation. The level of statistical significance was set at *p* < 0.05 for all analyses.

### 2.8. Ethical Issues

Ethical approval was received from the Karamanoğlu Mehmetbey University Social and Human Sciences Scientific Research and Publication Ethics Committee (Decision Date: 28 November 2023, Decision Number: 16-2023/286). Permission to conduct the study in the designated kindergarten was granted by the Provincial Directorate of National Education (Date: 15 January 2024, Number: 94320281). Participation in the study was voluntary. Therefore, before starting the study, the parents of the children who planned to be included in the sample group were informed about the aim and process of the study. Written consent was obtained from the parents, who voluntarily agreed to their children’s participation in the study, and verbal consent was obtained from the children. Given the sensitive nature of the study, a child safety procedure was established prior to data collection and was planned to be followed throughout the intervention and assessment process. If a child exhibited distress or anxiety, the session was paused, and the child was not required to continue. When necessary, psychosocial support was facilitated through the relevant school services. In the event of a disclosure or reasonable suspicion of child sexual abuse, the session was discontinued, the child’s immediate safety and emotional well-being prioritized, and repeated or suggestive questioning was avoided. In accordance with the mandatory reporting provisions of the Turkish Penal Code (Law No. 5237, Articles 278–280), such cases were to be reported without delay to the competent judicial or law-enforcement authorities (Public Prosecutor’s Office or police/gendarmerie), with referrals to appropriate child protection and psychosocial support services, including child advocacy centers, where appropriate. No disclosure or suspected case of child sexual abuse occurred during the study; therefore, activation of this reporting and referral pathway was not needed.

## 3. Results

The mean age of the parents included in the study was 33.34 ± 6.358 years (min = 23, max = 53), 90.6% were female, 62.5% were high school graduates, and 71.9% were housewives. All the families were nuclear families, and all defined their income level as less than their expenses. Among the children, 46.9% were 5 years old, 53.1% were girls, and 59.4% had one sibling. One child was found to have a metabolic disorder. For 59.4% of the participants, the number of people living in the household was 3 (Table 1).

The skewness and kurtosis values for several scale scores fell outside the expected range (±1.5), indicating deviations from normality. Therefore, nonparametric methods were used for pretest–posttest comparisons (Table 2).

**Figure 1:**
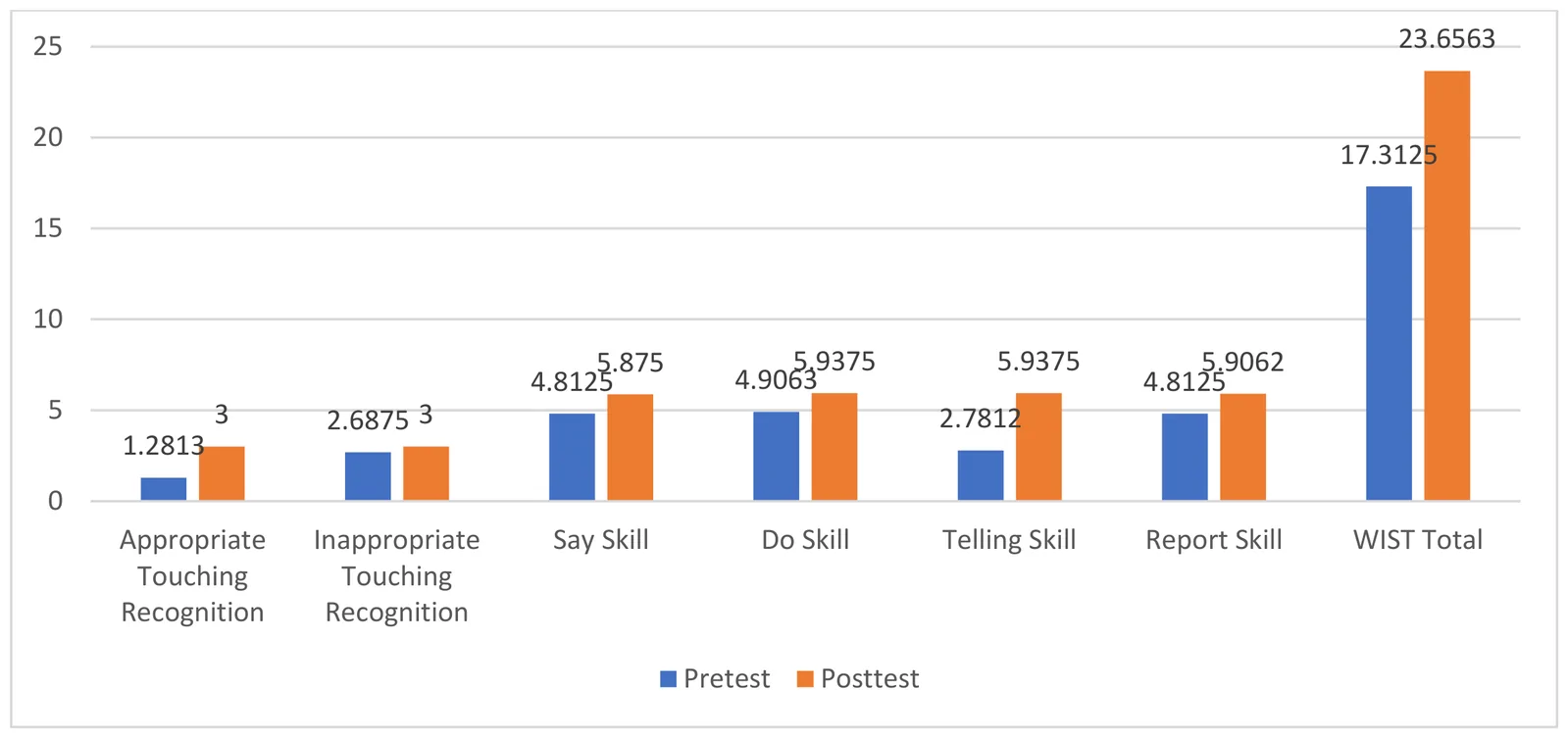
Bar chart of the pretest and posttest scores for the WIST.

A statistically significant difference was found between the children’s WIST total skill pretest and posttest scores (Z = −4.756, *p* < 0.001), with a mean change of 6.34 (95% bootstrap CI: 4.63–8.13). In addition, statistically significant differences were found between the children’s pretest and posttest scores on the WIST Appropriate Touch (Z = −4.364, *p* < 0.001; mean change = 1.72, 95% CI: 1.31–2.13), Inappropriate Touch (Z = −2.271, *p* = 0.023; mean change = 0.28, 95% CI: 0.09–0.50), Saying Skill (Z = −2.980, *p* = 0.003; mean change = 1.06, 95% CI: 0.53–1.63), Doing Skill (Z = −3.097, *p* = 0.002; mean change = 1.03, 95% CI: 0.53–1.56), and Telling Skill (Z = −4.741, *p* < 0.001; mean change = 3.16, 95% CI: 2.53–3.72). In the Wilcoxon signed-rank test, the effect size r, which indicates the magnitude of the difference, is interpreted as small when 0.1, medium when 0.3, and large when approximately 0.5 [29]. Accordingly, the effect size for the difference between children’s WIST total skill pretest and posttest scores was 0.84, indicating a large effect. The effect size for the difference between the pretest and posttest scores was 0.77 for the appropriate touch subscale (large effect), 0.40 for the inappropriate touch subscale (medium effect), and 0.84 for the telling skill subscale (large effect), whereas for the playing skill subscale and doing skill subscale, it was 0.53 and 0.55, respectively, indicating large effects. A statistically significant difference was found between children’s WIST Reporting Skill pretest and posttest scores (Z = −2.961, *p* = 0.003), with a mean change of 1.09 (95% bootstrap CI: 0.53–1.69) and an effect size of 0.52, indicating a large effect (Table 3 and Figure 1).

**Figure 2:**
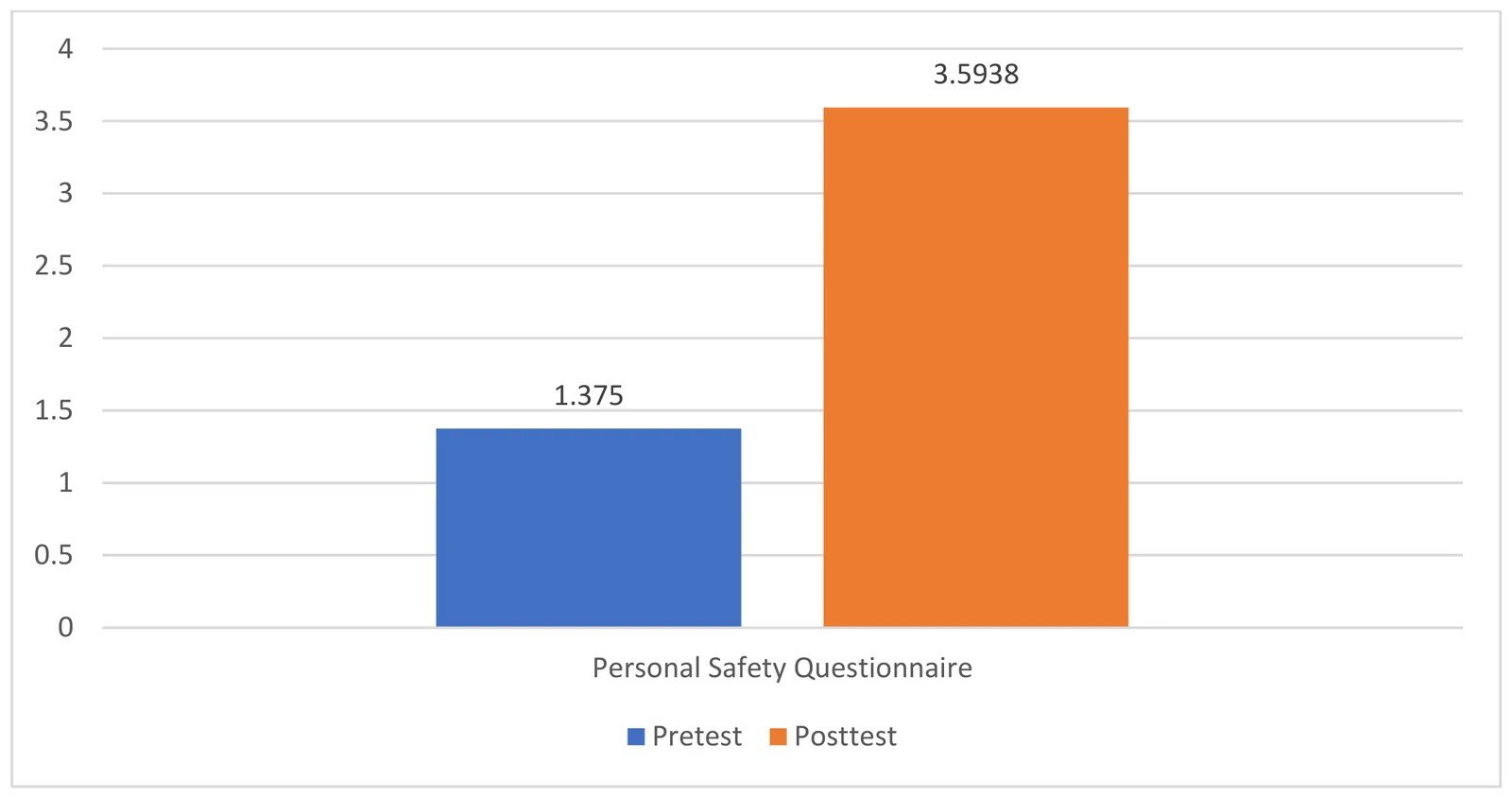
Bar chart of the pretest and posttest scores for the PSQ.

A statistically significant difference was found between the children’s PSQ pretest and posttest scores (Z = −4.913, *p* < 0.001; mean change = 2.22, 95% CI: 1.88–2.56). Accordingly, the effect size for the difference between children’s PSQ pretest and posttest scores was 0.87, indicating a large effect (Table 4 and Figure 2).

In addition, since the scores did not follow a normal distribution, the relationships between the children’s WIST total skill scores and PSQ posttest scores were examined via Spearman’s rank-order correlation. No relationship was found between the children’s WIST total skill scores and PSQ posttest scores (r_s_ = −0.034, *p* = 0.853, 95% CI: −0.312–0.312). However, given the restricted variability in posttest WIST scores due to clustering near the maximum possible values, this correlation should be interpreted with caution.

## 4. Discussion

Child sexual abuse is a critical public health concern. Interventions such as educating children about risks and strengthening their self-protection skills are needed, and their long-lasting psychological effects make them a priority for both children and society [11,30]. Prevalence rates in developing and non-Western low- and middle-income countries are substantially higher—and more concerning—than those reported in developed/Western countries. Therefore, there is a need for research on preventing child sexual abuse and evaluating related interventions in developing countries [31]. In this context, the current study investigated changes in the self-protection skills of children from families living in a region of Türkiye characterized by low socioeconomic status and high rates of violence following participation in the BST Program.

In the present study, children showed significant improvements in their posttest scores on both the Appropriate Touch subscale and the Inappropriate Touch subscale compared with the pretest. Their WIST total skill scores, as well as their Saying, Doing, Telling, and Reporting subscale scores, were also significantly higher at the posttest. Similarly, children achieved significantly higher posttest scores on the PSQ than pretest scores did. However, these improvements should be interpreted cautiously given the one-group pretest–posttest design and the absence of a control group. Therefore, the observed changes cannot be attributed solely to the BST Program, as maturation, repeated testing, or other external factors may also have contributed to the findings. Although only a limited number of studies have examined the effectiveness of school-based child sexual abuse prevention programs among children from low socioeconomic backgrounds, recent research supports our findings. For example, Kenny et al. (2022) evaluated the effectiveness of a school-based curriculum (Child Safety Matters) implemented in 2053 children aged 4–13 years (441 of whom were preschoolers) in public schools in low-income areas of Florida [32]. The researchers reported gains in children’s knowledge levels from the pretest to the posttest and reported significant differences at the *p* < 0.001 level between the pretest and posttest scores across all grade levels [32]. In Ecuador, Bustamante et al. (2019) investigated the effects of a 10-week program (I Have the Right to Feel Safe) delivered in primary schools serving predominantly low socioeconomic populations [33]. Children aged 7–12 years demonstrated notable gains in self-protection strategies, and these improvements were maintained over a 6-month follow-up. Their thematic analysis revealed that the greatest change occurred in children’s understanding that not all secrets should be kept [33]. Similarly, in Turkey, Kızıltepe et al. (2022) examined the effectiveness of the five-module programme I Am Learning to Protect Myself with Mika among preschool children in schools representing “low–middle” and “middle–high” socioeconomic levels [15]. The results revealed that the children in the intervention group gained significantly more knowledge and self-protection skills than did the children in the control group, and these gains persisted at the two-month follow-up. Interestingly, improvements were consistent across socioeconomic levels, indicating that children benefited equally regardless of background [15]. Although the school-based programs and methodologies used varied, these studies, together with the present findings, suggest that school-based sexual abuse prevention programs may support the development of knowledge and self-protection skills among children from low socioeconomic backgrounds.

Research suggests introducing child sexual abuse prevention programs when children are developmentally capable of learning the medical names of their genital organs [34] and as early as possible [11]. Indeed, a meta-analysis of school-based education programs in China revealed that, in terms of knowledge and skills, preschool children benefited more from the interventions than primary and middle school children [35]. Recent meta-analyses have also shown that interventions including more than three sessions tend to be more effective than those including three or fewer sessions [36] and that short-session programs addressing children’s socioemotional skills and self-blame are more effective [13]. The improvements observed in the self-protection skills of the children in this study group may be associated with the fact that the participants were preschool children and that the BST program implemented in the present study included the program components recommended above.

In the present study, a statistically significant improvement was also observed in the Reporting Skill subscale, with a large effect size. Current research emphasizes that school-based abuse prevention programs may benefit from active parental involvement to increase their effectiveness [13,36,37]. Çıtak Tunç et al. (2021) conducted a cross-sectional study in Türkiye with 92 preschool children aged 3–6 years and their parents to examine the effect of parent–child communication in BST on children’s self-protection skills [38]. The study demonstrated that BST delivered through parent–child discussions was effective in improving both Doing Skill and Reporting Skill [38]. These findings are consistent with the improvement in Reporting Skill observed in the present study and suggest that incorporating parents into future BST programs may further support the development and reinforcement of children’s reporting skills.

## 5. Conclusions

This study showed that participation in the seven-session Body Safety Training Program was associated with significant improvements in preschool children’s body safety knowledge and self-protection skills, including recognition of appropriate and inappropriate touching, saying “No,” getting away from unsafe situations, telling a trusted adult, reporting skills, and overall WIST total skill scores. These findings suggest that structured, developmentally appropriate body safety education may be a promising approach for supporting personal safety skills among preschool children from low-income families.

Given the one-group pretest–posttest design, the results should be interpreted as preliminary and should not be viewed as definitive evidence of intervention effectiveness. Nevertheless, the findings highlight the potential value of incorporating body safety education into preschool settings, particularly in socioeconomically disadvantaged communities.

## 6. Strengths, Limitations, and Future Research

Examining the effectiveness of the BST program in a low socioeconomic group, which represents an important risk factor for child sexual abuse, is one of the main strengths of this study. Other strengths of the study include the use of a short-term program consisting of seven sessions of approximately 20 min each that encourage children’s active participation and the evaluation of the program’s effectiveness through pretest and posttest measurements.

The study was limited by its single-site implementation, small sample size, low participation rate, and one-group pretest–posttest design. The low participation rate may have introduced selection bias, as participating children and families may have differed from those who did not participate, while the small sample size limits the generalizability of the findings. Although the achieved sample size (*n* = 32) exceeded the minimum sample size determined by the a priori power analysis (*n* = 26), the absence of a randomized control group limits causal inference regarding the effectiveness of the BST Program. Specifically, improvements observed between the pretest and posttest assessments cannot be attributed exclusively to the intervention, as maturation, repeated testing, or external events occurring during the study period may have contributed to the observed changes. Moreover, because the postintervention assessment was conducted only one day after completion of the BST program and no longer-term follow-up assessment was performed, the sustainability of the observed improvements over time could not be determined. A potential ceiling effect was observed for several WIST skill subscales at posttest, with scores clustering near the maximum possible values and showing restricted variability. This restricted variability may have limited the ability of these subscales to discriminate between participants after the intervention and may also have attenuated associations involving posttest WIST scores. Therefore, the magnitude of the observed intervention effects, including the large effect sizes, should be interpreted cautiously, and the nonsignificant association between the WIST total skill score and PSQ posttest score should also be interpreted in light of this restricted variability. In addition, the open-ended responses in the WIST skill subscales were scored by a single researcher according to the standardized scoring criteria; therefore, interrater agreement could not be assessed. Furthermore, although the BST Program was delivered according to the planned session content and structure, no formal intervention fidelity assessment (e.g., an independent observer or standardized fidelity checklist) was conducted. Therefore, adherence to the intervention protocol could not be independently verified. Additionally, the pretest and posttest assessments were administered by researchers who were also involved in implementing the intervention; however, the scoring of the open-ended WIST responses was performed by a single researcher. The assessors were not blinded to the assessment time point; therefore, expectancy or observer effects cannot be ruled out.

Future studies should examine the effectiveness of the BST Program for preschool children through adequately powered, multicenter randomized controlled trials with larger samples, in which participants are randomly assigned to intervention and control groups. Such designs would allow researchers to better distinguish the effects of the intervention from maturation, repeated-testing effects, and other external influences. Parental involvement should also be incorporated into future programs through feasible strategies tailored to low-income settings, such as brief parent education sessions scheduled alongside school activities, take-home parent–child activities, and flexible participation options. Integrating these activities into a routine school program may reduce participation barriers and facilitate sustained parental engagement. Follow-up assessments should also be conducted to evaluate the long-term sustainability of the intervention effects. It is recommended that nurses and other health professionals working with preschool children take an active role in implementing the BST Program, as their involvement could significantly contribute to the enhancement of children’s self-protection skills. Future studies should employ independent raters and report interrater reliability indices for rubric-based scoring while also incorporating systematic fidelity monitoring to ensure consistent implementation of the BST Program. Future studies should use independent, blinded outcome assessors to minimize potential measurement bias.

## Figures and Tables

**Table 1:** Demographic characteristics of parents and children.

Variables	Categories	*n*	%
Parent			
Gender	Female	29	90.6
	Male	3	9.4
Educational status	Primary	4	12.5
	Secondary	20	62.5
	University	8	25
Occupation	Housewife	23	71.9
	Self-employed	9	28.1
Family type	Nuclear	32	100
Economic status	Income is less than expenses	32	100
Child			
Age	4	11	34.4
	5	15	46.9
	6	6	18.8
Gender	Female	17	53.1
	Male	15	46.9
Number of siblings	0	4	12.5
	1	19	59.4
	2	8	25
	3	1	3.1
Presence of illness	Yes	1	3.1
	No	31	96.9
Household size	1	1	3.1
	2	6	18.8
	3	19	59.4
	4	6	18.8

**Table 2:** Assessment of the normality of scale scores.

Scales	Pretest		Posttest	
	Skewness	Kurtosis	Skewness	Kurtosis
Appropriate Touching Recognition	0.376	−1.4	*	*
Inappropriate Touching Recognition	−1.975	2.32	−5.657	32
Say Skill	−1.205	0.395	−3.795	13.227
Do Skill	−1.039	−0.464	−5.657	32
Tell Skill	0.760	−0.248	−5.657	32
Report Skill	−1.205	0.395	−4.430	20.144
WIST Total	−0.999	−0.042	−5.102	27.155
PSQ	−0.112	−0.543	−0.401	−1.967

PSQ: Personal Safety Questionnaire; *: Skewness and kurtosis could not be calculated for the appropriate touching recognition posttest scores because all participants obtained the same score.

**Table 3:** Comparison of Children’s WIST Pretest and Posttest Skill Scores.

Scales	Group	N	Mean	SD	Mean Rank	Z	*p*	r
Appropriate Touching Recognition	Pretest	32	1.28	1.20	0	−4.364	0.000	0.77
	Posttest	32	3.00	0.00	12.5			
Inappropriate Touching Recognition	Pretest	32	2.69	0.69	0	−2.271	0.023	0.4
	Posttest	32	3.00	0.00	3.5			
Say Skill	Pretest	32	4.81	1.73	0	−2.980	0.003	0.53
	Posttest	32	5.87	0.49	6			
Do Skill	Pretest	32	4.90	1.53	0	−3.097	0.002	0.55
	Posttest	32	5.93	0.35	6.50			
Telling Skill	Pretest	32	2.78	1.67	0	−4.741	0.000	0.84
	Posttest	32	5.93	0.35	14			
Report Skill	Pretest	32	4.81	1.73	0	−2.961	0.003	0.52
	Posttest	32	5.90	0.39	6			
WIST Total	Pretest	32	17.31	5.25	0	−4.756	0.000	0.84
	Posttest	32	23.65	1.45	15			

SD: Standard deviation, r: Effect size value; Z: Wilcoxon signed-rank test statistic.

**Table 4:** Comparison of children’s PSQ pretest and posttest scores.

Scale	Group	N	Mean	SD	Mean Rank	Z	*p*	r
Personal Safety Questionnaire	Pretest	32	1.38	0.83	0.00	−4.913	0.000	0.87
	Posttest	32	3.59	0.50	16.00			

SD: Standard deviation, r: Effect size value; Z: Wilcoxon signed-rank test statistic.

## Data Availability

The data presented in this study are provided upon reasonable request from the relevant author. Owing to ethical and confidentiality restrictions regarding child participants, the data are not publicly available.

## Abbreviations

The following abbreviations are used in this manuscript:

BST	Body Safety Training
WIST	What If Situations Test
PSQ	Personal Safety Questionnaire
